# The Influence of Anthocyanidin Profile on Antileishmanial Activity of *Arrabidaea chica* Morphotypes

**DOI:** 10.3390/molecules25153547

**Published:** 2020-08-03

**Authors:** Carla Junqueira Moragas-Tellis, Fernando Almeida-Souza, Maria do Socorro dos Santos Chagas, Paulo Victor Ramos de Souza, João Victor Silva-Silva, Ygor Jessé Ramos, Davyson de Lima Moreira, Kátia da Silva Calabrese, Maria Dutra Behrens

**Affiliations:** 1 Natural Products Department, Pharmaceutical Technology Institute-Farmanguinhos, Rio de Janeiro, Rio de Janeiro 21040-900, Brazil; msocchagas@gmail.com (M.d.S.d.S.C.); pvrs.pvrs@gmail.com (P.V.R.d.S.); ygorjesse@gmail.com (Y.J.R.); dmoreira@far.fiocruz.br (D.d.L.M.); mariabehrens@hotmail.com (M.D.B.); 2 Laboratório de Imunomodulação e Protozoologia, Instituto Oswaldo Cruz, Oswaldo Cruz Foundation, Rio de Janeiro, Rio de Janeiro 21040-360, Brazil; jvssilva89@gmail.com (J.V.S.-S.); calabrese@ioc.fiocruz.br (K.d.S.C.); 3 Postgraduate in Animal Science, Universidade Estadual do Maranhão, São Luís 65055-310, Maranhão, Brazil

**Keywords:** Bignoniaceae, *Leishmania amazonensis*, anthocyanidins, phytochemical profile, season, carajurin

## Abstract

*Arrabidaea chica* Verlot (crajiru) is a plant used in folk medicine as an astringent, anti-inflammatory, wound healing and to treat fungal and viral diseases such as measles chickenpox and herpes. *Arrabidaea chica* has several morphotypes recognized but little is known about its chemical variability. In the present study the anthocyanidin profile of *A. chica* morphotypes collected in two seasons (summer and winter) have been examined and their activity against *Leishmania* infection compared. High-performance liquid chromatography coupled to a diode-array detector (HPLC-DAD-UV) and by tandem mass spectrometry with electrospray ionization (ESI-MS/MS) were used for anthocyanidin separation and identification. Antileishmanial activity was measured against promastigote forms of *Leishmania amazonensis*. Multivariate analysis, principal component analysis (PCA) and Pearson’s correlation were performed to classify morphotypes accordingly to their anthocyanidin profile. The presence of 6,7,3′,4′-tetrahydroxy-5-methoxyflavylium (3′-hydroxy-carajurone) (1), carajurone (2), 6,7,3′-trihydroxy-5,4′-dimethoxy-flavylium (3′-hydroxy-carajurin) (3) and carajurin (4), and three unidentified anthocyanidins were detected. Two different groups were recognized: group I containing 3′-hydroxy-carajurone; and group II with high content of carajurin. Among anthocyanidins identified in the extracts, only carajurin showed significant statistical correlation (*p* = 0.030) with activity against *L. amazonensis*. Carajurin could thus be considered as a pharmacological marker for the antileishmanial potential of the species.

## 1. Introduction

*Arrabidaea chica* (Humb. and Bonpl.) Verlot (syn. *Fridericia chica*. L.G. Lohmann, which became the official name of the species) belongs to the Bignoniaceae family, which comprises of about 100 genera and 860 species [1]. This species is popularly known as crajiru, pariri and cipó cruz among other names [2,3,4]. The plant is listed as a natural dye, its leaves being used traditionally by indigenous peoples as a body paint [5,6]. It is also used in folk medicine as an astringent and anti-inflammatory agent and to treat skin wounds and ulcers, fungal infections and viral diseases like measles, chickenpox and herpes [7,8,9,10,11,12]. An infusion of the leaves may be used both orally and topically to cleanse wounds [13].

Several varieties of the species are described in the literature, among them: *A. chica* var. *acutifolia* DC. (syn. *Arrabidaea rosea* DC., *Temnocydia carajuru* M. and *Vasconcellia acutifolia* M.) with larger leaves (up to 15 cm in length), and a purplish red corolla and minor (up to 2 cm); *A. chica* var. *angustifolia*, with a smaller corolla, and smaller lanceolate leaves (up to 5 cm in length); *A. chica* var. *cuprea* (syn. *Bignonia cuprea* Cham.), again with smaller, narrow, short-obtuse-acuminate leaves and copper-colored reticulation on the lower face; *A. chica* var. *thyrsoidea* (syn. *Bignonia chica* HBK., *Bignonia thyrsoidea* DC.), with large leaves (up to 10 cm long and 6 cm wide), a larger panicle and a 3 cm corolla [7]. Little is recorded of the phytochemical variation that may accompany these varieties or of the relation between their metabolites and their biological activity [2].

According to Embrapa Amazonia Occidental at Brazilian Agricultural Research Company, in Manaus, there are at least three crajiru morphotypes, which are also recognized by local communities. They are called morphotypes I, II and III and have as a common characteristic the absence of a flowering period, since only collections of vegetative organs have been registered, which makes their taxonomic identification more difficult [14]. In addition to the three morphotypes of Manaus, a fourth one, which has bigger and brighter leaves than the others, called morphotype IV, is cultivated at the Oswaldo Cruz Foundation (Fiocruz) Atlantic Forest Campus near Rio de Janeiro.

**Figure 1:**
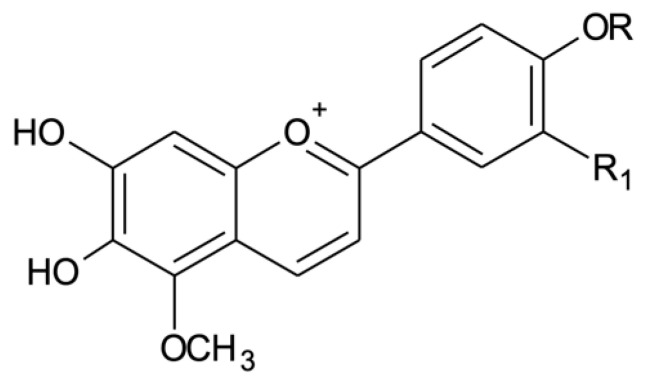
Chemical structures of the anthocyanidins described for *Arrabidaea chica*: (**1**) 3′-hydroxy-carajurone (R_1_ = OH, R = H); (**2**) carajurone (R_1_ = H, R = H); (**3**) 3′-hydroxy-carajurin (R_1_ = OH, R = CH_3_) and (**4**) carajurin (R_1_ = H, R = CH_3_).

*Arrabidaea chica* is characterized by the presence of 3-deoxy-anthocyanidins. Four of these anthocyanidins have been identified for this species in the literature. Carajurin stands out as the most important of them. First described by [5], carajurin appears as the main chemical marker of *A. chica* in several publications. The isolation of carajurin from leaves and flowers suggests that it was exclusive to the genus *Arrabidaea* and possibly limited to the species *A. chica* [15]. The author noted that further research within the genus would be necessary to confirm this conclusion. At present, there is no mention of the isolation of carajurin from another plant species, confirming the author’s suggestion that carajurin is the chemical marker for *A. chica* extracts. Studies by [16] and [17] described the extraction, isolation and chemical characterization of four 3-deoxyanthiocyanidins in *A. chica*: 6,7,3′,4′-tetrahydroxy-5-methoxy-flavylium (3′-hydroxy-carajurone) (**1**)**,** 6,7,4′-trihydroxy-5-methoxy-flavylium (carajurone) (**2**), 6,7,3′-trihydroxy-5,4′-methoxy flavylium (3′-hydroxy-carajurin) (**3**) and 6,7-dihydroxy-5,4′-dimethoxy-flavylium (carajurin) (**4**) (Figure 1). Anthocyanidins are chemically distinct from other types of flavonoids because they have a flavylium cation in their structure, which results in the visible absorption range for this class of compounds, from λ 469 to 492 nm [17].

Besides anthocyanidins, further studies of the constituents of *A. chica* report the occurrence of tannins [6,18], chalcones [19], polyphenols [20] and other flavonoids [18]. However, most of the research on *A. chica* does not define which variety or morphotype was being investigated. This lack of information is a serious failing because the differences observed in the chemical composition of the *A. chica* morphotypes lead to different biological responses. Previous work has demonstrated the leishmanicidal activity of *A. chica* against *Leishmania amazonensis*, the principal parasite responsible for cutaneous leishmaniasis [19]. This work aims to evaluate the influence of the morphotype and season of collection on the *A. chica* anthocyanidins profile and verify how this variation affects their leishmanicidal activity.

## 2. Results and Discussion

### 2.1. Characterization and Quantitation of Anthocyanidins (mg/g of Dry Extract) Expressed as Carajurin Content of Four A. chica Morphotypes

Since anthocyanidins are among the most important phytochemical constituents of *A. chica* [9], being pointed out as chemical markers of the species [5,15] we evaluated the influence of season of collection and morphotype on their content in several extracts (Table 1). The results of the quantitative determination of seven identified anthocyanidins (mg/g of dry extract calculated as carajurin content) are presented in Table 1.

Identification and peak assignment of anthocyanidins in all extracts was based on a comparison of their retention time (Rt), elution order and UV spectrum with published data [17]. Anthocyanidin 1–4 assignments were confirmed by mass spectral data obtained by direct infusion in the positive mode (tandem mass spectrometry with electrospray ionization (ESI-MS/MS); Supplementary Materials of Figure S1).

All compounds listed in Table 1 showed UV λ_max_ in the range of 474–485 nm, characteristic of anthocyanidins. Previous studies [16,17] had already described and characterized compounds **1**–**4** by their UV spectra. Carajurin was first described by Chapman in 1927 [5], however, until 1980 only UV data had been described for this compound [15].

**Figure 2:**
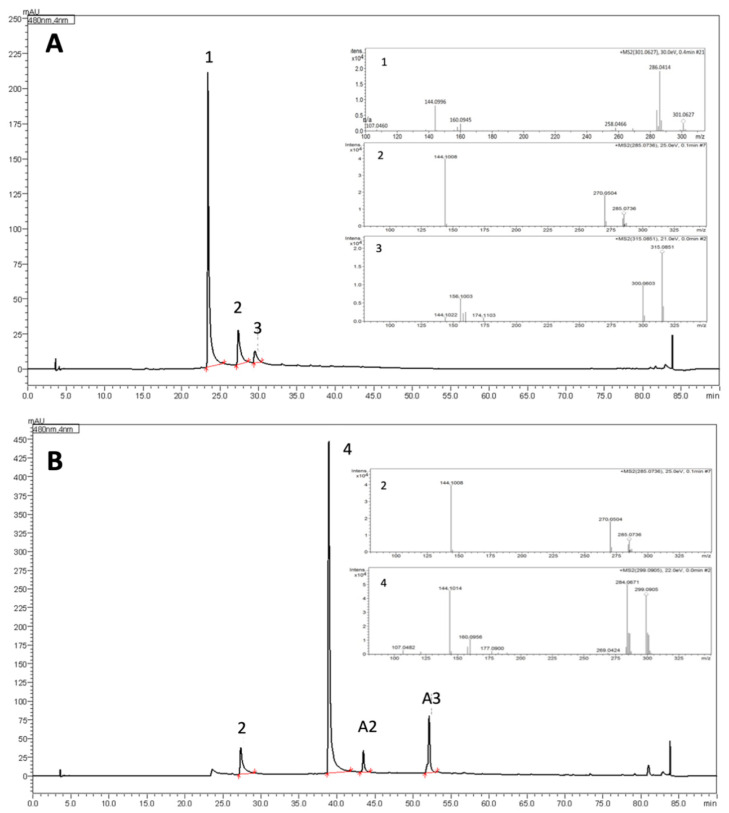
Chromatograms of morphotypes I (W) (**A**) and IV (S) (**B**) showing the elution order of anthocyanidins 1–4 and their respective mass spectra. **A**. Compound **1** (Rt = 23.5 min; M + [H]^+^ at *m*/*z* 301.0627; compound **2** (Rt = 27.4 min; M + [H]^+^ at *m*/*z* 285.0736 and compound **3** (Rt = 29.5 min; M + [H]^+^ at *m*/*z* 315.0851). **B**. compound **4** (Rt = 39.0 min; M + [H]^+^ at *m*/*z* 299.0905). Compounds **A2** and **A3** (Rt = 43.5 min and 52.1 min) showed M + [H]^+^ at *m*/*z* 317.0756 and *m*/*z* 287.0614, respectively.

Concerning the elution order, 3′-hydroxy-carajurone (6,7,3′,4′-tetrahydroxy-5-methoxyflavylium) (**1**) was the first to be eluted (Rt = 23.5 min) due to its more polar structural features (four hydroxyls groups). Carajurone (6,7,4′-trihydroxy-5-methoxy-flavylium) (**2**), which contains three hydroxyl groups, was eluted at Rt = 27.3 min followed by 3′-hydroxy-carajurin (6,7,3′-trihydroxy-5,4′-dimethoxy-flavylium) (**3**) (Rt = 29.5 min) that possess three hydroxyl groups and two methoxyl groups, and finally by carajurin (6,7-dihydroxy-5,4′-dimethoxy-flavylium) (**4**) (Rt = 39.0 min), the least polar of the four known anthocyanidins from *A. chica* (two hydroxyl and two methoxyl groups). This elution order is in agreement with the mobile (polar since acidified water and acetonitrile it was used in gradient) and stationary phases (silica-based C18 column, non-polar) used to proceed with the separation (Figure 2, Figures S2 and S3).

Another three anthocyanidins indicated as **A1** (Rt = 25.4 min), **A2** (Rt = 43.5 min) and **A3** (Rt = 52.1 min) were assigned in some morphotype extracts from this species (Table 1).

Table 1 shows significant differences among morphotypes concerning anthocyanidin mg/g of dry extract calculated as carajurin content (mean of triplicate) in the two analyzed seasons. Carajurin was found in morphotypes ACI and ACIV (S) and ACII (W), but not detected in the other morphotypes in either annual collection. Carajurone stood out as the only one found in all morphotypes in both seasonal collections. Compound 3′-hydroxy-carajurone was identified in the majority of analyzed extracts, but not in ACI and ACIV (S) and ACII (W), exactly those in which carajurin was found. This finding may indicate a probable correlation between the biosynthesis of these two 3-deoxyanthiocyanidins. Compound 3′-hydroxy-carajurin was present only in the extracts of morphotypes I and II (W) and in small concentrations, indicating that among the four anthocyanidins already characterized for *A. chica*, this is the least abundant.

Other anthocyanidins detected in this study, also seem to be present only in some extracts, in lower concentrations (**A2** and **A3** under 3.89; **A1** under 1.68 mg/g of dry extract), justifying the fact that they were not previously isolated and structurally elucidated.

**Figure 3:**
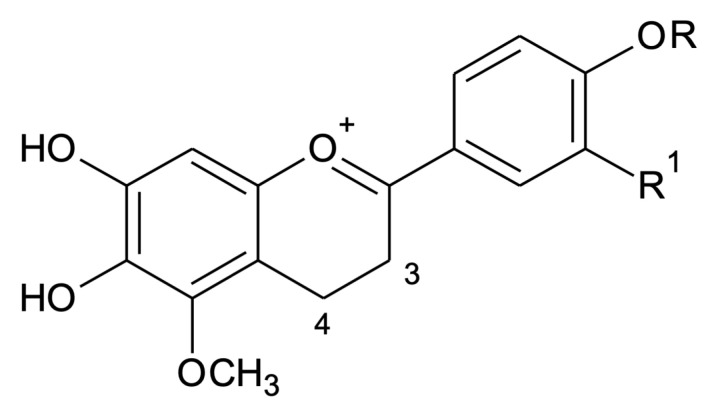
Proposed structures for **A1** (C_16_H_15_O_6_, *m*/*z* 303.1922, R_1_ = OH, R = H), **A2** (C_17_H_17_O_6_, *m*/*z* 317.0756, R = CH3, R_1_ = OH) and **A3** (C_16_H_15_O_5_, *m*/*z* 287.0614, R = R_1_ = H).

Positive ESI-MS/MS (Figure S4) data analyses were also performed to confirm the structures of the four known anthocyanidins in extracts of different morphotypes in two seasons. Compound **1** (3′-hydroxy-carajurone), showed experimental pseudo-molecular ion (M + [H]^+^) at *m*/*z* 301.0627 (calc. *m*/*z* 301.0717, ppm error (∆) = 29.8 ppm) compatible with the molecular formula C_16_H_13_O_6_. Compound **1** fragmentation pattern produced ions at *m*/*z* 286.0414 [(M) − CH_3_]^+^ and at *m*/*z* 256.0466 [(M) − CH_3_ − CO]^+^. The exact mass of the fragments was also compared to their calculated mass and showed ∆ of −5.59 and −25.96, respectively. These data are in agreement with 6,7,3′,4′-tetrahydroxy-5-methoxyflavylium. Compound **2** (carajurone) was confirmed by the experimental pseudo-molecular ion at *m*/*z* 285.0736, corresponding to the molecular formula C_16_H_13_O_5_ (calc. *m*/*z* of 285.0749; ∆ = −4.56), as well as by the mass fragments at *m*/*z* 270.0492 [(M) − CH_3_]^+^ (calc. *m*/*z* of 270.0533; ∆ = −15.44) and *m*/*z* at 242.0541 [(M − CH_3_ − CO)]^+^ (calc. *m*/*z* of 242.0584; ∆ = −17.76). Compound **3** (3′-hydroxy-carajurin) showed a pseudo-molecular ion at *m/z* 315.0851 (calc. *m*/*z* of 150.8740; ∆ = −7.29) corresponding to the molecular formula C_17_H_15_O_6_ as well as fragments with *m/z* 300.0588 (calc. *m*/*z* of 300.0639; ∆ = −16.99) and *m*/*z* 285.0355 (calc. *m*/*z* of 285.0404; ∆ = −17.19) that confirm the loss of two methyl groups (M − CH_3_]^+^_2_). The registered fragment at *m/z* 257.0409 (calc. *m/z* of 257.0455; ∆ = −17.89) is related to C=O loss. These data confirm compound **3** as 6,7,3′-trihydroxy-5,4′-methoxyflavylium. Compound **4** (carajurin) was confirmed by the pseudo-molecular ion at *m*/*z* 299.0905 (calc. *m/z* of 299.0924; ∆ = −6.35) corresponding to the molecular formula C_17_H_15_O_5_ as well as the fragments at *m/z* 284.0671 (calc. *m*/*z* of 284.0690; M - [CH_3_]^+^; ∆ = −6,68) and *m*/*z* 269.0441 (calc. *m*/*z* of 269.0455; M − CH_3_]^+^; ∆ = −5.20). In the mass spectrum for compound **4** a CH_3_ loss followed by C=O loss, was also observed producing *m*/*z* ion at 256.0722 (calc. *m*/*z* of 256.0741; ∆ = −7.41), followed by CH_3_ loss, generating *m/z* ion at 241.0491 (calc. *m*/*z* of 241.0506; ∆ = −6.22). Both the pseudo-molecular ion and the fragments described in this study for the **1**–**4** anthocyanidins are in agreement with those data previously published [14]. The anthocyanidins indicated as **A1**, **A2** and **A3** were detected in lower percentages and could not be identified, showing pseudo-molecular ions at *m/z* 303.1922 (C_16_H_15_O_6_), *m*/*z* 317.0756 (C_17_H_17_O_6_) and *m*/*z* 287.0614 (C_16_H_15_O_5_), respectively. These anthocyanidins had already been presented in a previous study [21] when the authors showed, in addition to the four anthocyanidins already known, three others with the same molecular masses as those presented here. The compounds **A1**, **A2** and **A3** showed respectively two more units of mass than the identified 3′-hydroxy-carajurone, 3′-hydroxy-carajurin and carajurone, which may suggest the presence of two more hydrogen atoms in their structures. We can suggest the hydrogenation of the carbons at positions 3 and 4 of the flavylium C-ring that would be compatible with the proposed structures (Figure 3). Further isolation and chemical characterization will be necessary to confirm the proposed structures.

### 2.2. Multivariate Analysis

**Figure 4:**
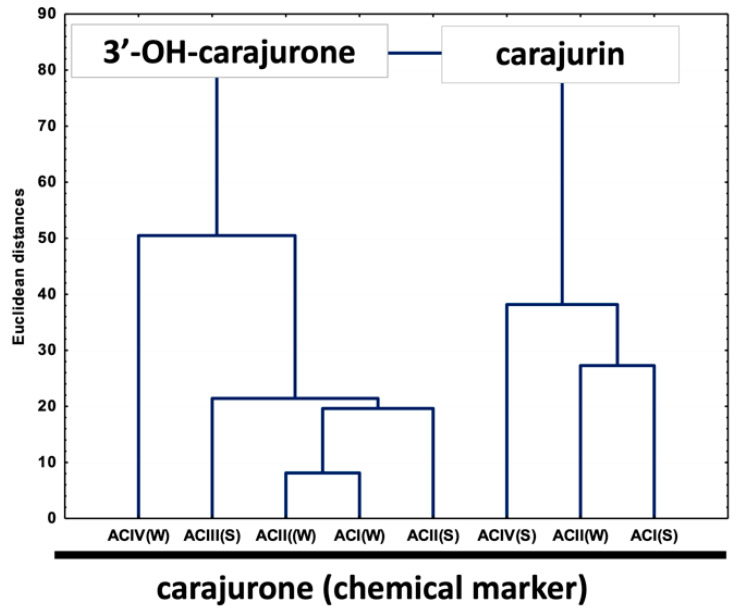
Unweighted air-group method with arithmetic average (UPGMA) dendrogram showing the similarity of chemical composition in anthocyanidins among eight extracts of *Arrabidaea chica* Verlot. 3′-OH-carajurone = 3′-hydroxy-carajurone.

Multivariate analysis based on the anthocyanidin composition of *A. chica* morphotypes and seasonal variation allowed the separation of the samples in two different groups (Figure 4). Group I is composed of extracts ACIV(W), ACIII(S), ACI(W), ACII(W) and ACII(S); and group II by extracts ACIII(W), ACIV(S) and ACI(S). The important variable for the group I discrimination is 6,7,3′,4′-tetrahydroxy-5-methoxyflavylium (3′-hydroxy-carajurone, **1**). The extracts of the morphotypes that compose group II are characterized by the high concentration of carajurin (**4**). Carajurone (**2**) was identified in all extracts and with less influence on the analysis of variance, so it can be chosen as a chemical marker for all these morphotypes and seasons.

**Figure 5:**
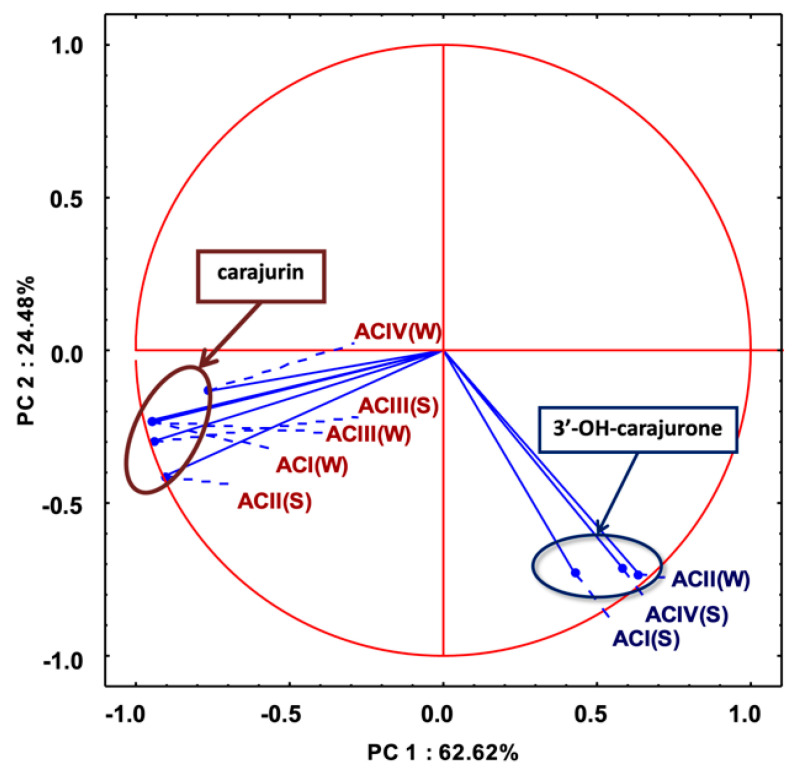
Principal component analysis of variation of chemical composition in anthocyanidins among eight extracts of *Arrabidaea chica* Verlot: scores plot. 3′-OH-carajurone = 3′-hydroxy-carajurone.

PCA analysis (considering the percentage content of anthocyanidins; Figure 5) showed two main components (with an eigenvalue greater than 1) explaining 91.6% of the total variance of the data, considering the scores plot to simplify the analysis of the results. This result confirms the formation of the two groups previously demonstrated in Figure 4, one composed by 6,7,3′,4′-tetrahydroxy-5-methoxyflavylium (3′-hydroxy-carajurone, **1**) and the other by carajurin (4) (Figure 5).

### 2.3. In Vitro Antileishmanial Activity

Leishmanicidal activity of four *Arrabidaea chica* Verlot morphotypes are shown in Table 2. Only three extracts showed values statistically different from the others: ACIII(W) and ACIV(W), which presented the highest values; and ACIV(S), which presented the lowest value and the better leishmanicidal activity among all analyzed extracts. All the other extracts presented similar IC_50_ values (Table 2).

There is little information about the antileishmanial activity of *A. chica*. One of the first reports did not evaluate the crude extract, but only five fractions from the hexane extract obtained by silica gel column chromatography with an increasing polarity gradient of *n*-hexane and ethanol. Three hexane extract fractions showed IC_50_ of 31.8, 152.2 and 198.5 μg/mL, while the other two fractions showed no activity against *L. amazonesis* [22]. Another report of leishmanicidal activity showed IC_50_ of 155.9 ± 0.118 μg/mL to crude ethanolic extract [19], obtained in the same way as we used, and presenting IC_50_ values similar to those found in this study. The plant material used in these two reports was collected in Brazilian Amazonian, but details about the botanical identification of variety or morphotype are missed for both studies.

Our results also showed that summer presented less variation than winter in IC_50_ values. Interestingly, the collection season shown in a previous study [16] was made in the summer season, corroborating the similarity in IC_50_ values observed with our data. There is a lack of information about the seasonality of the collection by [22]. The three extracts that presented variations in IC_50_ values showed an interesting profile. The two extracts that showed higher IC_50_ values when compared to the others were collected in winter, while the extract that showed lower IC_50_ values was collected in summer. Likewise, analyzing the multivariant analysis of the anthocyanidin composition and the PCA, it is seen that group II, which contained carajurin, has more extracts collected in summer, while group I, without carajurin, has more extracts collected in winter. Although we have little data to perform an appropriate statistical analysis, this finding suggests a possible influence of seasonality in enhancing the probability of *A. chica* morphotypes contain carajurin something that could be more explored in further studies.

Evaluating the ACIV morphotype extracts, we noticed that winter collection classified as group I presented one of the highest IC_50_, while summer collection classified as group II, rich in carajurin, had the lowest IC_50_ value. Seasonality is often associated with chemical composition, which can be directly related to the biological activity [23], so we performed the correlation analysis of anthocyanidin variation chemical composition with the IC_50_ value.

**Figure 6:**
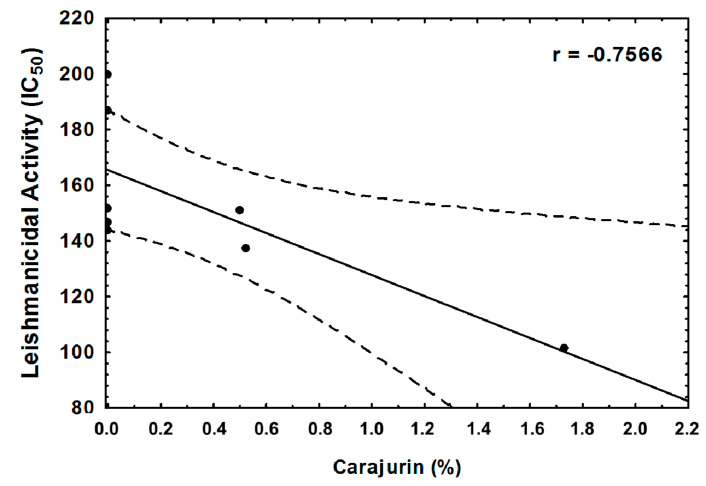
Correlation between carajurin obtained from *Arrabideae chica* and leishmanicidal activity against *Leishmania amazonensis*. *r* = −0. 7566 and *p =* 0.030 by the Pearson’s correlation analysis.

From the four chemical compounds identified in extracts, only carajurin showed a statistically significant correlation (*p* = 0.030), with it being directly related to a decrease in the IC_50_ value (Figure 6).

Carajurin has few biological activities reported in the literature. A bioguided fractionation of the methanol soluble part of the *A. chica* lipophilic extract using inhibition of NF-kB in Jurkat T cells as a target led to the isolation of four 3-desoxyanthocyanidins, among them carajurin [16]. *A. chica* extract, standardized for carajurone and carajurin, improved collagen organization and increased the quantity of dermatan sulfate of a partially transected tendon [24], however, there is a lack in the description of the plant variety or morphotype and the season of the year when the vegetal material was collected. Previous studies [19] did not identify the chemical constituents in the *A. chica* extract that demonstrated antileishmanial activity. The strong correlation observed between the carajurin presence and the increase in antileishmanial activity, indicate its potential as a possible marker for *A. chica* leishmanicidal activity to be explored in the future. It is worth mentioning that although the presence of a particular substance in an extract is generally associated with its biological activity, synergistic, antagonistic and additive effects among the compounds present in a plant extract should also be taken into account.

## 3. Materials and Methods

### 3.1. Plant Material Identification and Georeferencing

Aerial parts of *A. chica* Verlot morphotypes ACI, ACII, ACIII and ACIV cultivated at Fiocruz Atlantic Forest Campus, were collected, pressed and herborized in March 2016, according to [25]. Subsequently, the samples were deposited at the Botanical Collection of Medicinal Plants (CBPM) of Farmanguinhos/Fiocruz. Botanist Marcus Felipe Oliveira da Silva carried out species identification by using a stereomicroscope model TIM-2T (OPTON) and consulting analytical keys and specialized taxonomic literature. The plant material was compared with those deposited at the Herbarium of the Botanical Garden of Rio de Janeiro (RB) and CBPM. The sample images were scanned with ScanMaker 1000XL Plus (Microtek, Anaheim, CA, USA). The coordinates for the georeferencing of the specimens are in degrees decimals WGS 84. The registration data of all the samples as well as the images are in the virtual bank of CBPM (Brahms software, V2.1, Waltham, MA, USA). Voucher specimens were deposited at the CBPM under the numbers 665, 666, 667 and 668. The georeferencing of the four morphotypes of *A. chica* were ACI (S22.9407° W43.4046°); ACII (S22.9406° W43.4046°), ACIII (S22.9405° W43.4047°) and ACIV (S22.9406° W43.4047°).

### 3.2. Seasonal Collection, Extract Preparation and Isolation of Carajurin

Leaves of each identified morphotype (ACI-ACIV) were collected in two seasons, summer and winter. Leaves were dried at 60 °C with air circulation, ground and submitted to maceration in ethyl alcohol-water 70/30 (v/v) for seven days. The extracts obtained were filtered through filter paper, concentrated under reduced pressure at 30–40 °C in a rotary evaporator until complete evaporation of the solvent, packaged in amber bottles and stored at −20 °C until use. Extracts were named according to the morphotype and the season of collection. Those corresponding to leaves collected between December and March, (summer), received the code (S) and those collected between June and August, (winter), received the designation (W). To the isolation of carajurin, ACIV (S) extract was filtered and evaporated to dryness under reduced pressure at 30–40 °C. The reddish residue (15 g) was fractioned by liquid–liquid partition, with *n*-hexane (3 × 200 mL), dichloromethane (3 × 200 mL), ethyl acetate (3 × 200 mL) and *n*-butanol (3 × 200 mL). Dichloromethane fraction (ACDF, 4 g) was successively chromatographed on Sephadex LH-20 (Sigma-Aldrich, St. Louis, MI, USA) using methanol as eluent to afforded an anthocyanidin identified by direct infusion electrospray ionization mass spectrometry, ^1^H- and ^13^C-NMR spectrometry, as carajurin. Carajurin purity was evaluated by HPLC-DAD-UV as 98%.

Carajurin ^1^H-NMR (400 MHz-CDCl_3_-d6) d: 6.98 (d, 1H, H-3, *J* = 7.8Hz); 7.99 (d, 1H, H-4, *J* = 7.8 Hz); 7.01 (d, 2H, H-3′and H-5′, *J* = 8.8 Hz); 7.89 (d, 2H, H-2′ and H-6′, *J* = 8.8 Hz); 6.53 (s, 1H, H-8); 3.90 (s, 3H, 4′-O-CH_3_); 4.10 (s, 3H, 5-O-CH_3_). ^13^C NMR (100 MHz-CDCl_3_-d6) d: 158.90 (C-2); 102.62 (C-3); 133.76 (C-4); 135.02 (C-5); 139.93 (C-6); 176.82 (C-7); 98.61 (C-8); 156.86 (C-9); 118.16 (C-10); 123.43 (C-1′); 127.68 (C-2′); 114.77 (C-3′); 162.50 (C-4′); 60.42 (C-5′); 55.58 (C-6′). Positive ESIMS *m*/*z*: 299.0905 [M + H]+ (calc. *m*/*z* of 299.0924; ∆ = −6.35), *m*/*z* 284.0671 M − [CH_3_]^+^ (calc. *m*/*z* of 284.0690; ∆ = −6.68); *m*/*z* 269.0441 M − [CH_3_] − [CH_3_]^+^ (calc. *m*/*z* of 269.0455; ∆ = −5.20); *m*/*z* 256.0722 (M − [CH_3_] − [C=O]^+^) (calc. *m*/*z* of 256.0741; ∆ = −7.41); 241.0491 M − [CH_3_] − [CH_3_] − [C=O]^+^ (calc. *m*/*z* of 241.0506; ∆ = −6.22) (Figures S5 and S6).

### 3.3. Instrumentation and Chromatographic Conditions

#### 3.3.1. High-Performance Liquid Chromatograph Coupled with a Diode-Array UV-Vis Detector (HPLC-DAD-UV)

Chromatographic analyses were performed on a high-performance liquid chromatograph coupled with a diode-array UV-Vis detector (HPLC-DAD-UV), using a Shimadzu Nexera XR^®^ liquid chromatograph coupled to a Shimadzu, Kyoto, Japan, UV detector with the diode array SPDM20A, equipped with a CBM20A controller, DGU20A degasser, LC20AD binary pump, CTO20A oven and SILA20A auto-injector. A Shimadzu LabSolutions Software Version 5.3 (Shimadzu, Kyoto, Japan) was used to analyze chromatograms. DAD analysis was applied to select the optimized wavelength of anthocyanidins in this study. In a full-scan experiment, chromatograms at 480 nm show the maximum wavelength (λ_max_) for the anthocyanidins. Combinations of acidified ultrapure water (pH 3.0, with anhydrous acetic acid, Merck, Darmstadt, Germany) (A) and acetonitrile (HPLC grade, Tedia, Rio de Janeiro, Brazil) (B) were used as the mobile phase (initially 5% A rising to 95% in 80 min). HPLC column was silica-based C18 (250 mm × 4.6 mm i.d. × 5 μm particle size, ODS Hypersil, Thermo, Waltham, MA, USA). The oven was set at 50 °C and the injection volume was 10 μL for all analyses.

#### 3.3.2. Preparation of Extracts Samples

A total of 1000 μL of acetonitrile: methanol (both HPLC grade, Tedia) mixture (75:25; v/v) was added to 10 mg of extracts, previously weighed in a 4 mL vial. The vial was sealed and the sample was sonicated for 10 min with occasional swirling. Posteriorly, the sample was vortexed to mix thoroughly, followed by filtered through a 0.45 μm PTFE filter (Merck Millipore, Darmstadt, Germany) before further analyses into an HPLC vial.

#### 3.3.3. Quantification of Anthocyanidins Using Carajurin as the Standard

Quantification of anthocyanidins was performed using carajurin as the external standard. Since there are no standards available for all anthocyanidins, their content was expressed in milligrams of carajurin by grams of dry extract. Stock solution of the isolated carajurin (98%, chromatographic determined) was prepared as 200 μg/mL in acetonitrile:methanol (both HPLC grade, Tedia) mixture (75:25; v/v) in volumetric flasks. Six concentrations of work solutions were done on the day for analytical curve preparation (20; 40; 60; 100; 150 and 200 μg/mL) (Figure S7). The solutions were filtered in a 0.45 μm PTFE filter (Merck Millipore, Darmstadt, Germany) before analysis by HPLC-DAD-UV. Injections of 10 μL were performed in triplicate to obtain the analytical curve from the areas corresponding to the peaks of carajurin. Analytical curve (20–200 μg/mL) of the standard was constructed based on the UV-Vis signal at 480 nm to better selectivity: carajurin content (μg/mL) = (Abs (mAu) + 661228)/48694; R^2^ = 0.9993). Carajurin and other anthocyanidins amounts (mg/g of dry extract) were calculated and expressed as carajurin content.

#### 3.3.4. Tandem Mass Spectrometry with Electrospray Ionization (ESI-MS/MS)

ESI-MS/MS was recorded in a Bruker Ion trap amazon SL mass spectrometer. Anthocyanidins profile identification of each *A. chica* extract (1 μg/mL in HPLC grade methanol, Tedia, Brazil) was made by direct infusion in the positive ionization mode (ESI^+)^. The operating conditions were 1 μL/min infusion, 3.0–4.0 kV capillary voltage, 100 °C temperature source and cone voltage of 20–40 V. Mass spectra were recorded and interpreted by Bruker Compass Data Analysis 4.2. ESI-MS/MS was obtained for the anthocyanidin ions using collision energies ranging from 15 to 35 eV.

### 3.4. Parasites

Promastigote forms of *L. amazonensis* (MHOM/BR/76/MA-76), obtained from a human case of diffuse leishmaniasis, and characterized by isoenzyme [26] and lectin techniques [27], were maintained in Schneider’s Insect Medium (Sigma, USA) supplemented with 10% fetal bovine serum (Cultilab, Campinas, Brazil), penicillin (100 U/mL) and streptomycin (100 μg/mL; Sigma, St. Louis, MO, USA), incubated at 26 °C.

### 3.5. Activity Against Promastigote Forms

Promastigote forms of *L. amazonensis* (10^6^ parasites/mL) from a 3–5 day-old culture, were placed in 96-well plates with different concentrations of *A. chica* extracts (500–1.95 μg/mL, obtained by serial dilution 1:2), in a final volume of 100 μL per well, for 72 h. Wells without parasites were used as blank and wells with only parasites used as the control. The viability of the parasites was evaluated by counting viable parasites in a Neubauer chamber using optical microscopy on the 40× objective. The data were normalized according to the formula: % survival = sample counting/control counting × 100. The results were used to calculate the 50% inhibition of parasite growth (IC_50_). Amphotericin B was used as the reference drug.

### 3.6. Statistical Analysis

The IC_50_ were obtained from a nonlinear regression curve of the concentration log versus the normalized response. The numerical results were expressed as mean ± standard deviation and were organized into tables. Comparison between IC_50_ values was performed by Kruskal–Wallis and Dunn’s multiple comparisons test. Analyses were performed with the GraphPad Prism 6.0 software (GraphPad Software, San Diego, CA, USA). Differences were considered significant when *p* < 0.05. Chemometric studies were performed in order to understand covariance and to identify the relationship between the chemical composition of anthocyanidins present in extracts and the seasonal variations by principal component analysis routines (PCA) and hierarchical cluster analysis (HCA). A matrix composed by seven per eight counters (anthocyanidins × extracts) was constructed with the criteria of anthocyanidin content in the extracts obtained from the HPLC-DAD-UV analysis (the total amount of each anthocyanidin was obtained in a relative percentage from the signal area). Score graphs were drawn for PCA. The unweighted pair group method using the arithmetic averages (UPGMA) method and Euclidean distance was used for HCA. Correlation analysis between leishmanicidal activity and anthocyanidin content (relative percentage) in the extracts was performed using the Pearson’s correlation coefficient (*r*). Statistica 10 (StartSoft Inc., Tulsa, OK, USA) for Windows was used.

## 4. Conclusions

In conclusion, the analysis of *A. chica* extracts shows that anthocyanidin profiles change dramatically according to the morphotype and season. Only carajurone was identified in all analyzed extracts with variation between 1.67 ± 0.005 and 7.16 ± 0.080 mg/g of dry extract. By this mean carajurone can be the chemical marker of these morphotypes. The activities observed against promastigote forms of *L. amazonensis* suggest that carajurin content may be influencing the antileishmanial activity of *A. chica* and in this case could be related as a biological marker of the species. This assumption, however, needs to be confirmed in future studies.

## Figures and Tables

**Table 1:** Quantification of anthocyanidins (mg/g dry extract) calculated as carajurin content in four morphotypes of *Arrabidaea chica* Verlot (ACI-IV) collected in summer (S) and winter (W), and their retention times (Rt).

Compounds	Rt (min)	Anthocyanidin Content (mg/g of Dry Extract Calculated as Carajurin)							
		Summer				Winter			
		ACI	ACII	ACIII	ACIV	ACI	ACII	ACIII	ACIV
**1**	23.5		3.36 ± 0.020	9.64 ± 0.094	-	7.75 ± 0.066	-	9.92 ± 0.071	3.69 ± 0.041
**A1**	25.4	1.49 ± 0.002	-	-	-	-	-	-	1.68 ± 0.005
**2**	27.3	7.16 ± 0.080	1.67 ± 0.005	2.11 ± 0.003	3.54 ± 0.039	2.72 ± 0.006	2.51 ± 0.005	2.59 ± 0.015	2.53 ± 0.003
**3**	29.5		-	-	-	1.85 ± 0.005	-	1.75 ± 0.005	-
**4**	39.0	5.96 ± 0.010	-	-	17.26 ± 0.119	-	5.23 ± 0.022	-	-
**A2**	43.5	2.44 ± 0.005	-	-	2.10 ± 0.006	-	1.86 ± 0.001	-	2.38 ± 0.018
**A3**	52.1	2.10 ± 0.007	-	-	3.89 ± 0.022	-	2.63 ± 0.007	-	1.75 ± 0.010

Note: Values are expressed as the mean ± SD (*n* = 3, see experimental). Compounds: **1**. 3′-hydroxy-carajurone; **2**. Carajurone; **3**. 3′-hydroxy-carajurin and **4**. Carajurin. A1–A3 = unidentified anthocyanidins.

**Table 2:** Leishmanicidal activity of *Arrabidaea chica* Verlot extracts against promastigote forms of *Leishmania amazonensis* treated for 72 h.

Samples	IC_50_ (μg/mL)
ACI(S)	150.8 ± 0.064 ^a^
ACI(W)	146.7 ± 0.059 ^a^
ACII(S)	152.0 ± 0.043 ^a^
ACII(W)	137.7 ± 0.063 ^a^
ACIII(S)	144.3 ± 0.061 ^a^
ACIII(W)	199.9 ± 0.079 ^b^
ACIV(S)	101.5 ± 0.064 ^c^
ACIV(W)	187.2 ± 1.357 ^b^
Amphotericin B	0.37 ± 0.129 ^d^

Data represent mean ± SD of at least three independent experiments made in triplicate. IC_50_: inhibitory concentration of 50% parasites; (S): summer and (W): winter. Different letters in IC_50_ value column represent statistical difference between values after Kruskal–Wallis analysis and Dunn’s multiple comparison test (*p* < 0.05).

## Supplementary Materials

The following are available online. Figure S1: Full Scan-Positive and ESI-MS spectra of four *Arrabideae chica* morphotypes collected at summer (S) and winter (W); Figure S2. HPLC Profiles detected at 480 nm. Chromatograms of morphotypes I–IV of *Arrabidaea chica* collected in summer (S); Figure S3. HPLC Profiles Detected at 480 nm. Chromatograms of morphotypes I–IV of *Arrabidaea chica* Verlot collected in winter (W); Figure S4. ESI-MS/MS for the anthocyanidin ions (**1**–**4**) from *Arrabideae chica* using collision energies ranging from 15 to 35 eV; Figure S5. ^1^H-NMR spectrum (400 MHz) of carajurin (4); Figure S6. ^13^C NMR (100 MHz) spectrum of carajurin (4); Figure S7. Analytical curve (20–200 μg/mL) of the carajurin (4).
