# Supplementary material for: The Influence of Anthocyanidin Profile on Antileishmanial Activity of Arrabidaea chica Morphotypes
Source: Molecules. 2020 Aug 3;25(15):3547. doi: 10.3390/molecules25153547 (PMC7435800; doi:10.3390/molecules25153547)

Supplementary data

Figure S1. Full Scan - Positive and ESI-MS spectra of four *Arrabideae chica* morphotypes collected at summer (S) and winter (W)

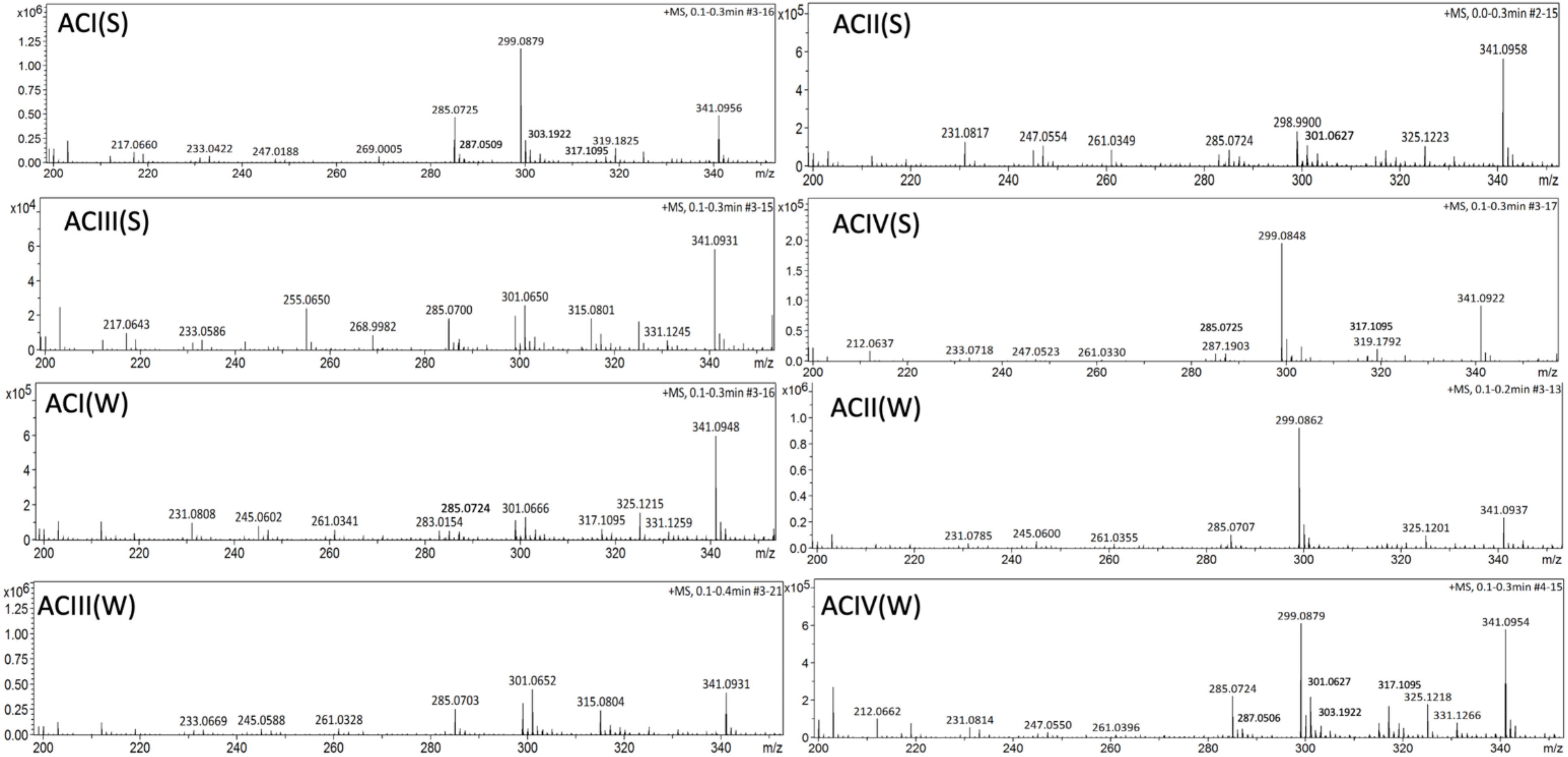

Figure S2. HPLC Profiles detected at 480 nm. Chromatograms of morphotypes I – IV of *Arrabidaea chica* collected in summer (S)

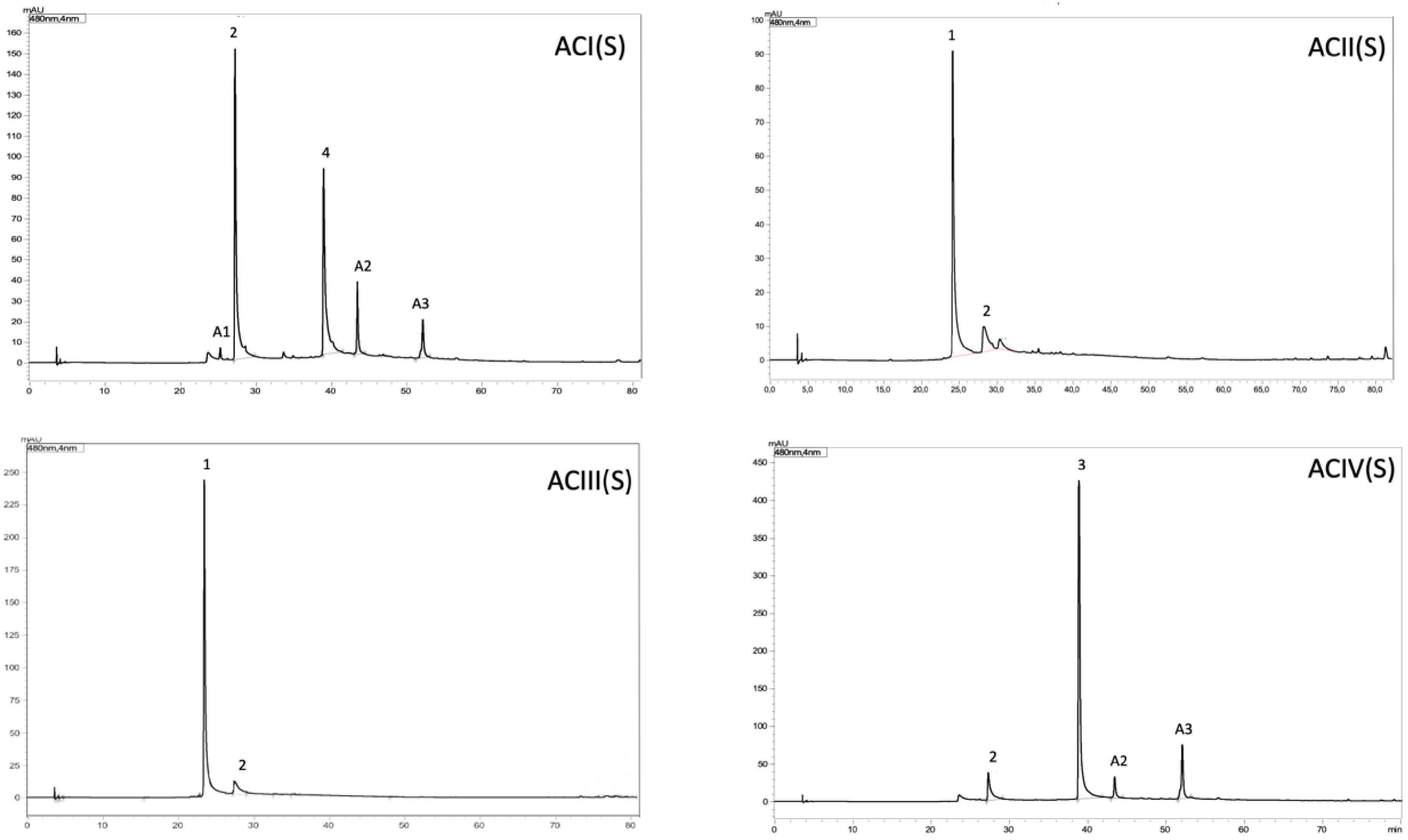

Figure S3. HPLC Profiles Detected at 480 nm. Chromatograms of morphotypes I – IV of *Arrabidaea chica* Verlot collected in winter (W)

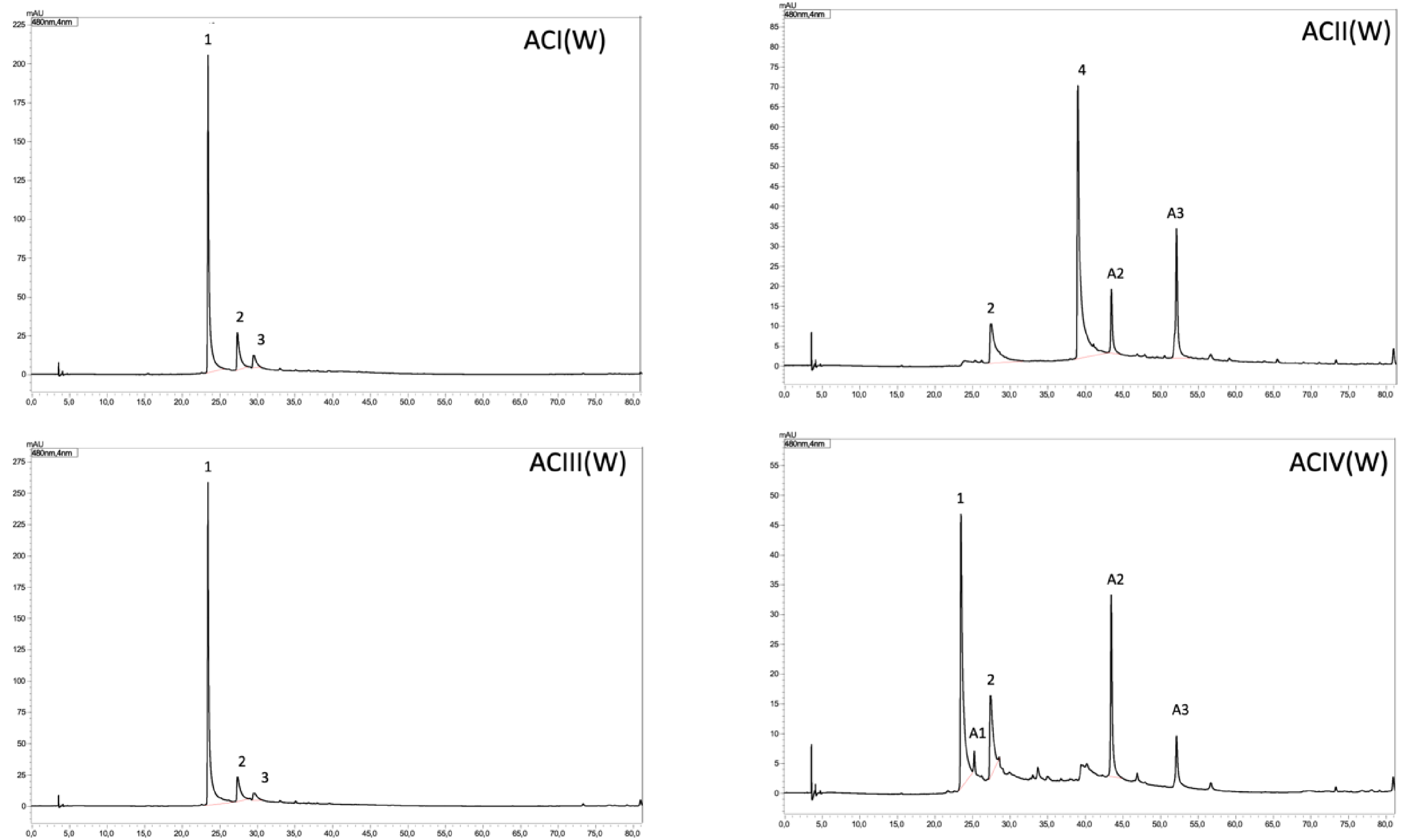

Figure S4. ESI-MS/MS for the anthocyanidin ions (**1-4**) from *Arrabideae chica* using collision energies ranging from 15 to 35 eV.

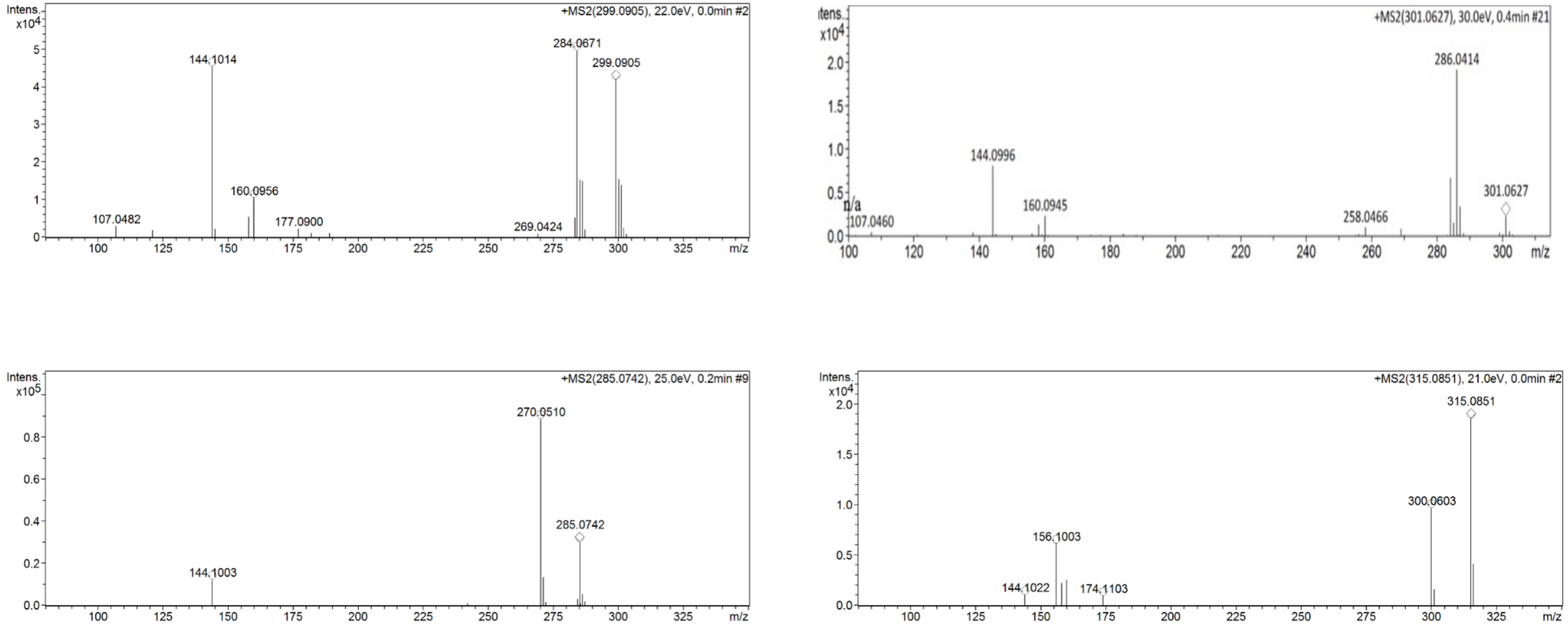

Figure S5. ¹H NMR spectrum (400 MHz) of carajurin (4)

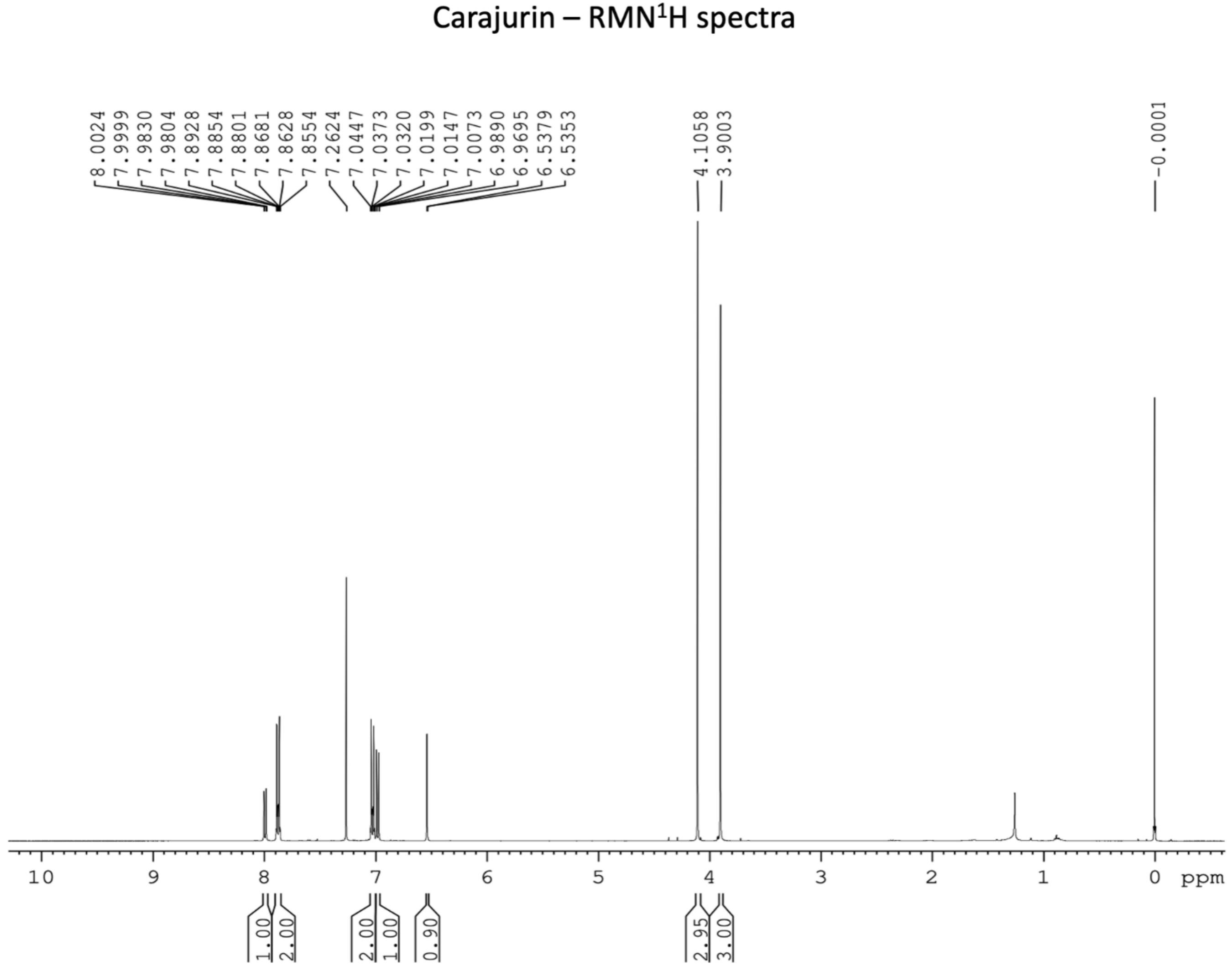

Figure S6. ^{13}C NMR (100 MHz) spectrum of carajurin (4)

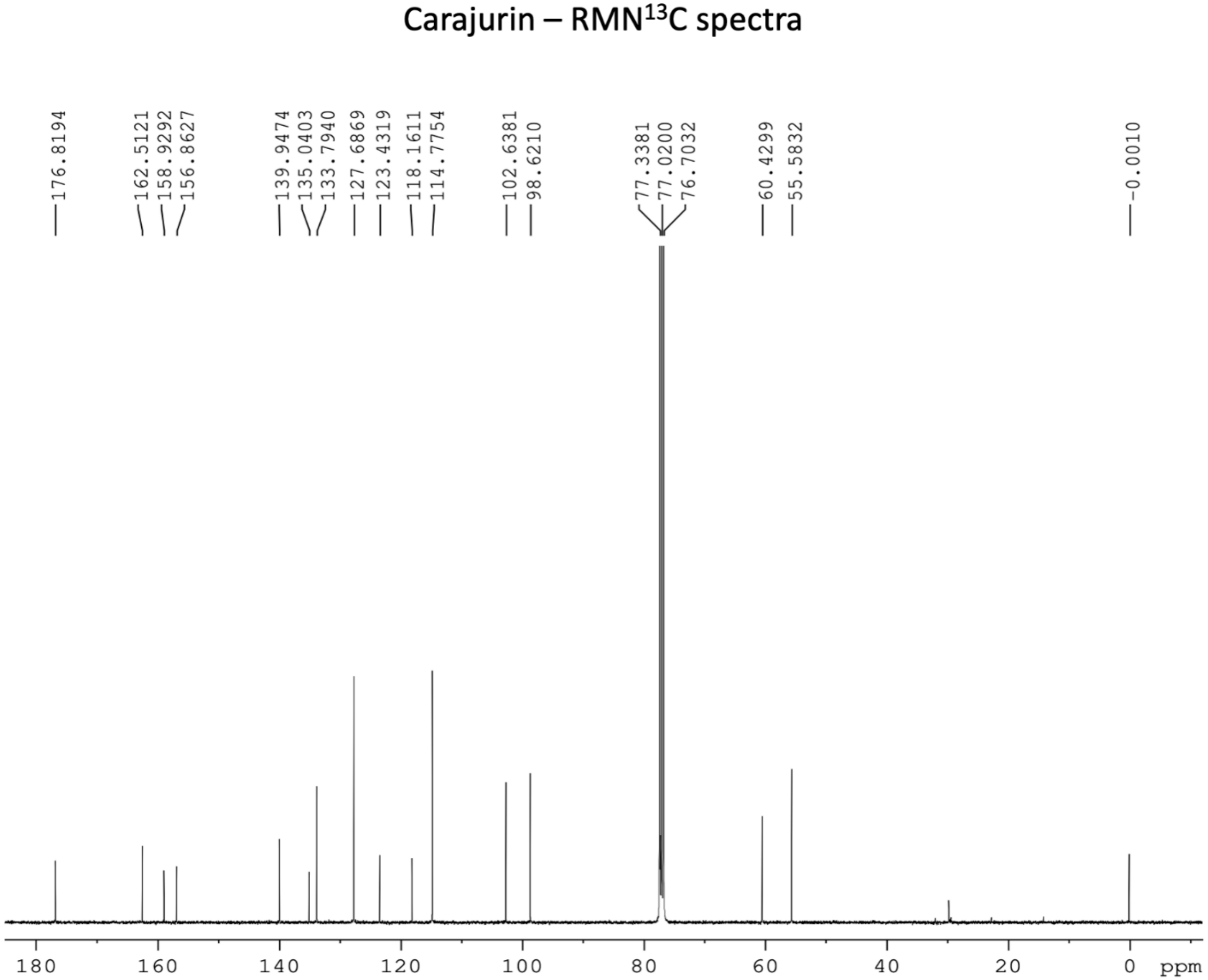

Figure S7. Analytical curve (20 – 200 μg/mL) of the carajurin (4).

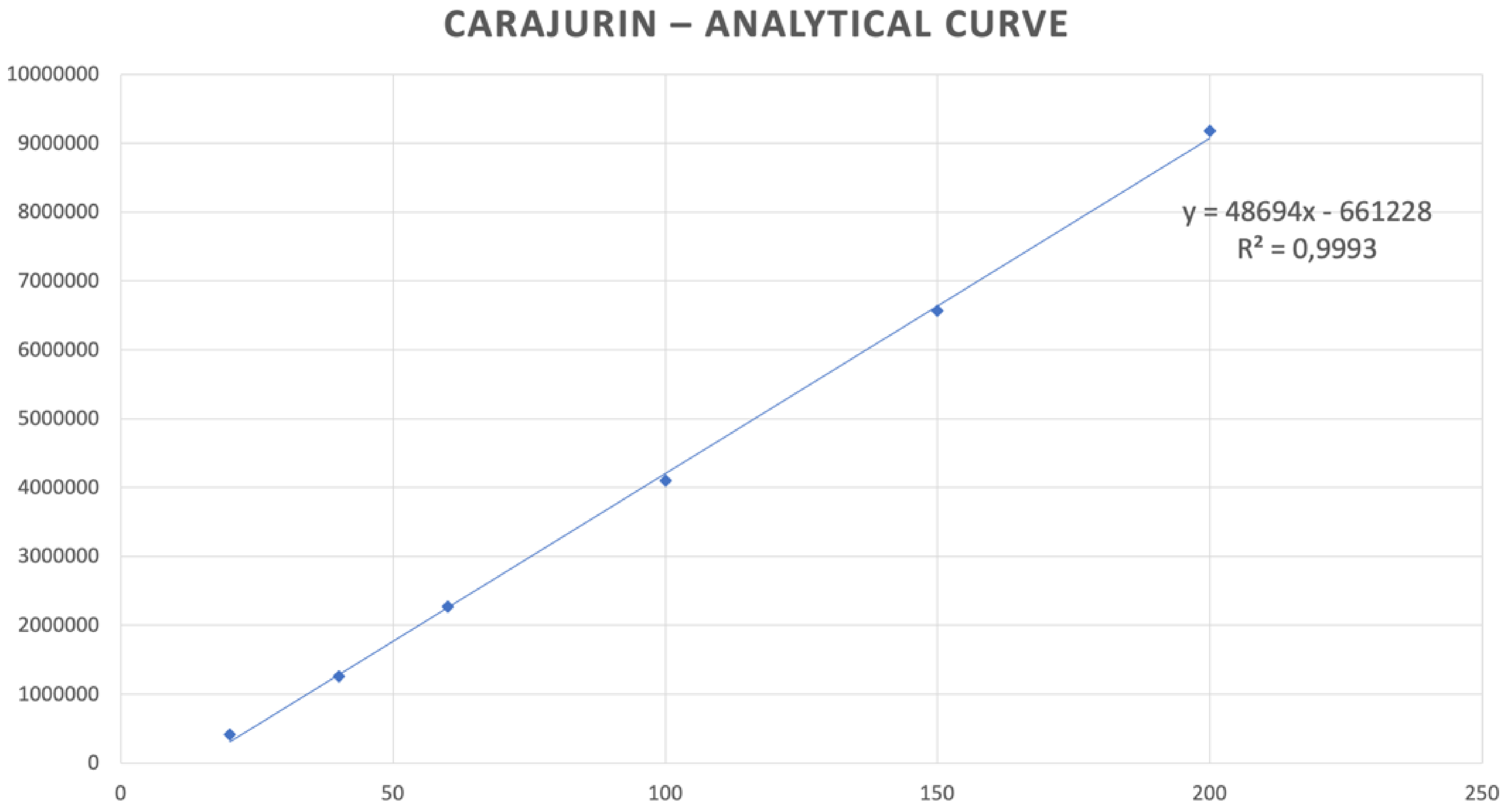

Supplement: Supplementary file 1 [file molecules-25-03547-s001.pdf]
